# Hyperdilute Calcium Hydroxylapatite for the Treatment of Perioral Rhytids: A Pilot Study

**DOI:** 10.1093/asjof/ojae021

**Published:** 2024-04-05

**Authors:** Michael Somenek

## Abstract

**Background:**

The perioral region plays a crucial role in facial aesthetics and is susceptible to age-related changes, such as wrinkles and fine lines due to its dynamic nature. Type I collagen is crucial in providing structural integrity and resilience to the skin. Calcium hydroxylapatite (CaHA-CMC) is a widely used dermal filler whose particles stimulate fibroblastic responses within the skin. When diluted, CaHA-CMC has emerged as a useful treatment for collagen stimulation.

**Objectives:**

The objective of the study is to evaluate the efficacy and safety of hyperdilute CaHA-CMC at a 1:3 ratio, specifically administered in the perioral area, to assess its impact on deep rhytids and overall skin quality.

**Methods:**

Females aged 40 to 70 years with moderate-to-severe wrinkles in the perioral region based on a validated wrinkle scale were injected with hyperdilute CaHA-CMC throughout the perioral region at 2 separate injection intervals (Weeks 1 and 8). This was followed by an injection of hyaluronic acid (CPM-HA22.5) into the perioral region at Week 16. The primary endpoint was a ≥1-point improvement from baseline on the wrinkle grading scale.

**Results:**

Twelve female participants were treated. Investigator and patient ratings based on the 5-point Merz perioral/lip wrinkle grading scale showed at least 1 grade improvement in 83% of the patients with a *P*-value of .0156. A majority of both investigators and patients rated their lip wrinkles as improved in appearance compared with their baseline.

**Conclusions:**

Hyperdilute CaHa-CMC at a 1:3 dilution may be a safe and effective treatment for improving the depth and overall appearance of perioral rhytids.

**Level of Evidence: 4:**

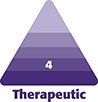

The perioral region plays a crucial role in overall facial aesthetics. This area is susceptible to age-related changes, including collagen degradation and elastin breakdown, and is considered particularly high risk for volume loss and dynamic rhytid formation, especially in females.^1^ These changes not only impact physical appearance but also have implications for psychological well-being and overall quality of life.^2^

Effectively addressing wrinkles and signs of aging in the perioral region presents a unique set of challenges due to its dynamic nature and the characteristics of its thick skin envelope. The mouth is in constant motion, engaging in activities, such as talking, eating, and a wide range of facial expressions, including lip pursing.^1,3^ Consequently, the repeated movements and muscle contractions in this area result in skin folding and stretching, ultimately leading to the development of wrinkles and fine lines over time. Furthermore, the perioral region exhibits a thicker skin structure than other facial areas, making it more resistant to various topical treatments and noninvasive interventions to reduce wrinkles and signs of aging. As the skin envelope naturally deteriorates with age, the continuous and persistent traction from mimetic facial muscles in this dynamic area contributes to and accentuates the pronounced depth of rhytids in the perioral region.^2,4^

Calcium hydroxylapatite (CaHA-CMC; Radiesse, Merz North America, Inc., Raleigh, NC), a widely utilized dermal filler, consists of biodegradable microspheres of CaHA-CMC suspended within an aqueous carboxymethyl cellulose gel carrier.^5^ Once injected, these biodegradable particles begin stimulating histiocytic and fibroblastic responses within the skin, acting as a scaffold to foster the formation of new tissue by promoting the development of collagen and elastin, essential elements for skin health and quality.^6^ This regeneration of neocollagenesis in the treated area can lead to notable improvements in skin laxity and overall skin quality. Consequently, these effects contribute to sustained aesthetic enhancement, providing patients with long-lasting rejuvenation that has been reported to last from 12 months to >3 years.^5-7^ The plane of injection is important for both nodule prevention and skin regeneration.

Collagen, specifically Type I, is crucial in providing structural integrity and resilience to the skin.^8^ Following the injection of CaHA-CMC, fibroblasts adhere to the CaHA-CMC microspheres, and through mechanical activation (ie, mechanotransduction), trigger fibroblasts to produce collagen Types I and III, elastin, proteoglycans, angiogenesis, and neovascularization. This intricate process stimulates dermal remodeling, consistent with the physiological progression of collagen Type I gradually replacing collagen Type III during the healing process.^9^ The synthesis of new collagen can commence as early as 4 weeks postinjection and has been observed to persist for up to 18 months after treatment, with collagen Type I being the predominant collagen present after 6 months.^6^

Undiluted CaHA-CMC is ideal for volume enhancement due to its highly viscoelastic nature.^9-11^ However, when diluted with lidocaine and/or saline, it exhibits newfound versatility, allowing for superficial injections and enhanced diffusion of CaHA-CMC particles throughout the dermis, effectively spreading wider and deeper. This technique has been used in the decolletage with a significant increase in dermal thickness and improved skin mechanical properties, including elasticity and pliability.^7^ The dilution ratio employed plays a pivotal role: lower ratios can achieve a combination of volume augmentation and dermal remodeling, whereas higher ratios focus more on regeneration without the volumizing effect.^9^ Many studies have meticulously examined the expression of collagen Types I and III in the dermis following the injection of CaHA-CMC at varying dilutions. Consistently, the findings have unveiled statistically significant increases in collagen production.^9,12^ Consequently, hyperdiluted CaHA-CMC has emerged as a useful treatment for collagen stimulation in areas less amenable to volumetric augmentation. This application effectively improves skin quality, augments skin elasticity, and initiates the process of angiogenesis, further accentuating multifaceted rejuvenation.^13^

CaHA-CMC is currently approved to address moderate-to-severe wrinkles and restore soft-tissue volume loss, both in the facial and hand regions. More recently, the utilization of diluted and hyperdiluted CaHA-CMC through subdermal injections has emerged as a valuable approach for enhancing the skin’s laxity, quality, elasticity, firmness, and reducing superficial wrinkles, thereby achieving an overall improvement in appearance.^14^ Although existing evidence supports the effectiveness of hyperdiluted CaHA-CMC in stimulating collagen production and enhancing skin quality, further research is needed to validate these findings comprehensively. Moreover, additional standardized information is needed on treatment protocols and injection techniques, thereby providing clinicians and patients with more comprehensive insights and guidance.

The aim of the present study is to evaluate the efficacy and safety of hyperdilute CaHA-CMC at a 1:3 ratio, specifically administered in the perioral area, to assess its impact on deep rhytids and overall skin quality. Despite the growing interest in this novel, off-label use of CaHA-CMC, limited literature support has resulted in only preliminary guidelines being available.^9,12^ This is the first study to our knowledge evaluating the use of hyperdilute CaHA-CMC for the treatment of perioral rhytids.

## METHODS

The study was approved by the Sterling IRB, and written informed consent was acquired from all patients. Twelve participants were enrolled in a prospective study conducted at a single center private practice in Washington, DC, by the primary author between August 2022 and May 2023. All participants were screened to ensure that they met all inclusion criteria, and none of the exclusion criteria before enrollment in the study. Participants recruited were not pregnant, fully understood the requirements and needs to comply with the study testing, and volunteered their willingness to discontinue any other antiaging topical or parenteral treatments for the duration of the study.

**Figure 1.**
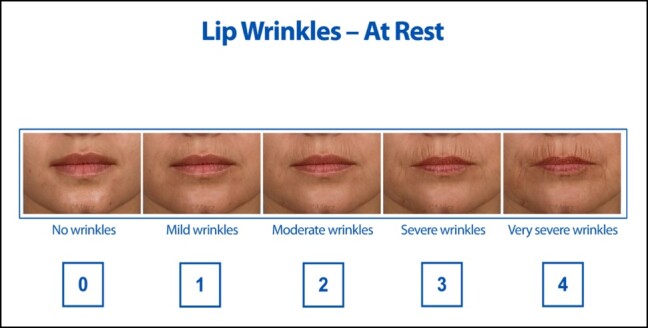
A 5-point Merz scale used to determine the severity of wrinkles in the perioral region. Image used with permission from Merz, Frankfurt, Germany.

Inclusion criteria were females aged 40 to 70 years with moderate-to-severe (Grade 2 or 3) wrinkles in the perioral region based on the 5-point Merz scale (Figure 1). The development of perioral rhytids is a problem specifically found in females; therefore, the inclusion of males was not deemed to be necessary. Exclusion criteria included any injectable, energy-based, or chemical peel to the perioral region in the past year, any autoimmune disease, and pregnancy or breastfeeding.

The rhytid score was determined based on a comparison of a series of 5 standardized photographs showing wrinkles of increasing depths, with Grade 1 being the mildest and Grade 4 being the deepest, in a static position.

Patients were injected with CaHA-CMC 1.5 mL at a dilution of 1:3 with 4.5 cc of sterile water into the perioral region at Day 0 and Week 8 (Visits 1 [V1] and 2 [V2], respectively) with a 25G 1.5 inch needle. For the technique, CaHA-CMC was injected subdermally using a fanning technique to cover the entire cutaneous perioral region that contained perioral rhytids. Average injection volumes were 3.8 cc for the entire perioral region.

At Visit 3 (Week 16), participants were injected with hyaluronic acid (CPM-HA22.5; Merz Aesthetics, Raleigh, NC) to address any remaining perioral rhytids. The study concluded with Visit 4 at Week 20, 4 weeks after the injection of CPM-HA22.5. During each visit, a review of adverse events and concomitant medications was carried out. A 2D photographic imaging and VISIA skin analysis were performed along with the patient and investigator Global Aesthetic Improvement Scale (GAIS), and patient and investigator rating on the Merz perioral rhytid scale.

Aesthetic improvement of perioral rhytids was rated by the patients and investigators, using the 7-graded GAIS from “very much worse” to “very much improved.”

The primary endpoint was a ≥1-point improvement from baseline on the 5-point Merz perioral/lip wrinkle grading scale evaluated by patients and investigators at 8, 16, and 20 weeks posttreatment (V2, V3, and V4, respectively).

The secondary endpoint of the study was to assess the clinical efficacy evaluated by patients and investigators at 8, 16, and 20 weeks posttreatment using the GAIS.

## RESULTS

A total of 12 female participants aged 51 to 70 years (average age 62.75 years) and Fitzpatrick skin Types I to III were treated in the study with 1:3 hyperdiluted CaHA-CMC. The average follow-up period is 6 months with a range of 5 to 7 months. All patients completed the entire duration of the study. Investigator ratings based on the 5-point Merz perioral/lip wrinkles grading scale showed at least 1 grade improvement in 83% of the patients (Table 1). Comparing the change from V1 to V4 using a signed-rank test (nonparametric test), there was a *P*-value of .0156, supporting that the difference between the first and last visit is greater than zero (ie, improved). The observed mean difference was 0.82 (95% CI 0.23-1.32).

**Table 1.** Investigator Response Differences Between V1 (baseline) and V4 (20 weeks)

Patient ID	V1-V4
1	0
2	1
3	0
4	1
5	0
6	2
7	1
8	1
9	2
10	1
11	1
12	1

**Figure 2.**
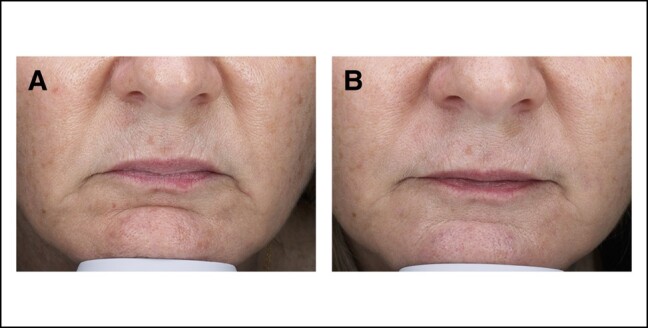
(A) A 52-year-old female at baseline, V1. Before injection with hyperdilute CaHA-CMC. (B) The same patient at V3 (16 weeks), after 2 injections of hyperdilute CaHA-CMC. CaHA-CMC, calcium hydroxylapatite.

**Figure 3.**
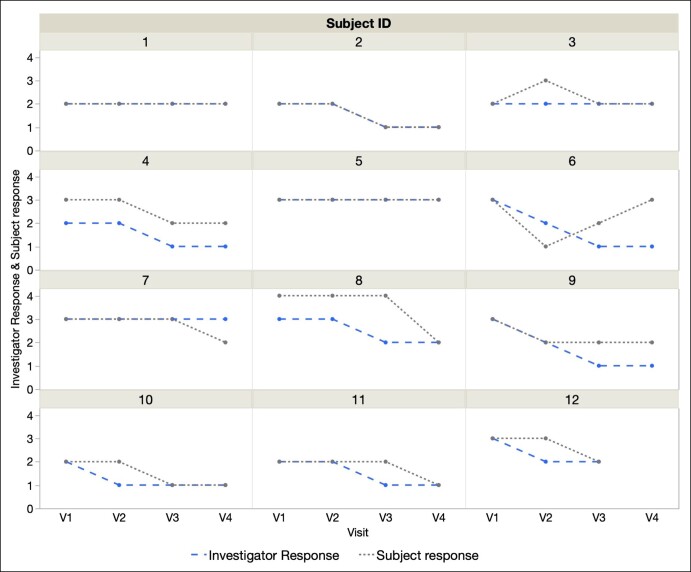
Investigator and patient responses over visits by patient ID. V1, baseline; V2, 8 weeks; V3, 16 weeks; V4, 20 weeks.

A majority of patients (75%) rated their lip wrinkles as improved between V1 and V4. Figure 2B shows 1 patient's response to hyperdilute CaHA-CMC, demonstrating a reduction in the depth and severity of her perioral rhytids when compared with baseline in Figure 2A. Patient 6 reported a decrease from severe wrinkles at V1 to mild wrinkles at V2 only to return to the baseline severe score by V4. All other patients either had no change (2 patients) or improved to at least 1 point by V3 (Table 2). This was the follow-up from the second injection of hyperdilute CaHA-CMC. Figure 3 demonstrates that both investigator and patient responses were similar longitudinally across all visits, with the exception of Patient 6.

**Table 2.** Patient Self-Rating Differences Between V1 (baseline) and V4 (20 weeks)

Patient ID	V1-V4
1	0
2	1
3	0
4	1
5	0
6	1
7	1
8	2
9	1
10	1
11	1
12	1

**Figure 4.**
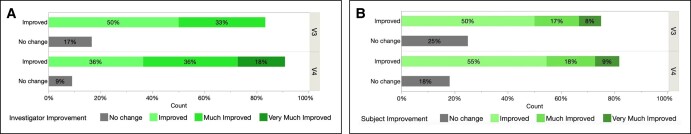
(A) Investigator and (B) patient Global Aesthetic Improvement Scale scores at V3 (16 weeks) and V4 (20 weeks) from baseline.

**Figure 5.**
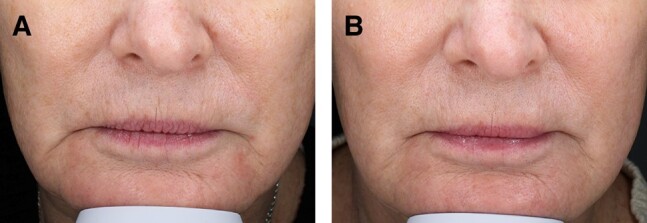
(A) A 64-year-old female at baseline, V1. Before injection with hyperdilute CaHA-CMC. (B) The same patient at V4 (20 weeks), after 2 injections of hyperdilute CaHA-CMC and 1 injection of CPM-HA22.5. CaHA-CMC, calcium hydroxylapatite.

The GAIS was used at 3 visits (V2, V3, and V4) to compare to the baseline observed at V1. By V2, the investigator rated all but 1 patient with improvement in appearance as compared to the initial V1 appearance. Thus, 91% were rated to be improved, whereas 55% were much or very much improved from the hyperdilute CaHA-CMC injection alone. By V2, the majority of patients felt that there was an improvement in appearance when compared with the initial visit (V1) appearance. By V4, following the injection of CPM-HA22.5, both patient and investigator GAIS scores improved to a greater degree (Figure 4). Figure 5B, representing V4 of 1 patient after injection of CPM-HA22.5, shows improvement in the overall appearance of the perioral region when compared with V1 (Figure 5A). The only adverse event that was documented was bruising postinjection, and it was categorized as mild in all instances.

## DISCUSSION

The perioral area is a unique anatomical region of bony and soft-tissue elements subject to dynamic forces that can result in complex rhytid formation and soft-tissue atrophy. Factors contributing to this include the effects of sun exposure, smoking and other environmental factors, hyperkinetic action of the orbicularis sphincter muscle, and genetics. There is very little quantitative evidence that exists for the directed treatment of this region. Some effective treatments have included skin remodeling through ablative resurfacing, deep chemexfoliation, and dermabrasion.^15,16^ However, downtime associated with these procedures can be substantial, and recovery can be complicated by persistent erythema, dyspigmentation, and scarring, especially in darker Fitzpatrick skin types.^17^ Efforts have focused on developing minimally invasive and low recovery approaches to treat perioral aging.^18^

The results of this prospective pilot study confirm that the use of hyperdilute CaHA-CMC at a 1:3 dilution is effective for improving the depth and overall appearance of perioral rhytids. There was at least a 1 grade improvement compared with baseline observed in 75% of both patient and investigator scores. Scores further improved after the injection of CPM-HA22.5 to address any residual perioral rhytids that had not yet responded to the hyperdilute CaHA-CMC. The injection of CPM-HA22.5 was incorporated into this study for a couple reasons. The effects of a collagen stimulator can take time to see a clinical result; in many cases, it takes 2 to 3 months after the injections have been performed. In the aesthetic patient, most are looking to see a more expedited result with a treatment while potentially waiting for the effects of the collagen stimulator to further enhance this area. To allow participants to have the most optimized appearance by the end of the study, CPM-HA22.5 allowed us to further soften any residual perioral rhytids that remained.

Patient selection is important because of the complex and dynamic nature of the perioral region. The perioral region can be characterized by many different changes, including volume loss, depth of perioral rhytids, skin dyspigmentation, and variances in the amount of dynamic motion and strain. Not all of these concerns are appropriate for treatment with hyperdilute CaHA-CMC. The depth of the rhytids may also play a role in how they respond to the collagen-stimulating effect of hyperdilute CaHA-CMC, as the deeper rhytids may be more resistant to showing a potential clinical result for the patient. These deeper rhytids may necessitate more treatment sessions or combination therapy with energy-based devices.^14^

Although hyperdilute CaHA-CMC has been used in other areas of the face and body with success, this is the first study to evaluate its application to the perioral region. Adverse events were limited to mild pain and transient bruising, similar to adverse events observed in other injectable studies.^19^ CaHA-CMC acts by supplementing the dermis with a scaffold to foster the formation of new tissue by promoting the development of collagen and elastin, essential elements for skin health and quality.^20^ Hyperdilution allows the CaHA-CMC microspheres to be more evenly distributed throughout the tissues, potentially providing the basis for why this is a safe and efficacious application for treating perioral rhytids.

Limitations to this study include the small population size. This was a pilot study to assess the efficacy and safety of hyperdilute CaHA-CMC in the perioral region. Additional studies with a larger population size should be considered now that preliminary data demonstrating safety and improvement with the chosen dilution and technique have been provided. Given the complex dynamics of the perioral region, further evaluating these results to assess for improvement in dynamic strain as well as improvement in the depth of the perioral rhytids would be helpful. The main objective of the study was to evaluate the effectiveness of hyperdilute CaHa-CMC to improve perioral rhytids. The addition of CPM-HA22.5 after the results of hyperdilute CaHa-CMC were evaluated is a noted weakness of this study. Although GAIS scores were calculated separately, the primary author understands that the addition of CPM-HA22.5 is a confounding variable. Further limitations include the use of a single evaluator and the limited length of the study.

## CONCLUSION

This study demonstrated that the use of hyperdilute CaHA-CMC injected around the perioral region may be safe and effective at reducing the depth and overall appearance of perioral rhytids. A comprehensive approach is frequently needed to address the age-related changes in the perioral region. Various degrees of volume loss, collagen degradation, and elastin breakdown can contribute to the depth and severity of perioral rhytid formation. This study attempts to enhance the current literature that exists regarding the use of hyperdilute CaHA-CMC by assessing its effectiveness in a region on the face that has yet to be published. The power of a collagen stimulator for the treatment of perioral aging should not be underestimated. Additional studies may be able to further elucidate how we can effectively apply this treatment for the long-term correction of perioral aging.
